# Urban Metabolic Analysis of a Food-Water-Energy System for Sustainable Resources Management

**DOI:** 10.3390/ijerph16010090

**Published:** 2018-12-30

**Authors:** Ming-Che Hu, Chihhao Fan, Tailin Huang, Chi-Fang Wang, Yu-Hui Chen

**Affiliations:** 1 Department of Bioenvironmental Systems Engineering, National Taiwan University, No. 1, Sec. 4, Roosevelt Road, Taipei 10617, Taiwan; mchu@ntu.edu.tw (M.-C.H.); cfwang@ntu.edu.tw (C.-F.W.); 2 Department of Urban Planning, National Cheng Kung University, No. 1, University Road, Tainan City 70101, Taiwan; tailinhuang@mail.ncku.edu.tw; 3 Department of Agricultural Economics, National Taiwan University, No. 1, Sec. 4, Roosevelt Road, Taipei 10617, Taiwan; yhc@ntu.edu.tw

**Keywords:** urban metabolism, food-water-energy, system dynamics simulation

## Abstract

Urban metabolism analyzes the supply and consumption of nutrition, material, energy, and other resources within cities. Food, water, and energy are critical resources for the human society and have complicated cooperative/competitive influences on each other. The management of interactive resources is essential for supply chain analysis. This research analyzes the food-water-energy system of urban metabolism for sustainable resources management. A system dynamics model is established to investigate the urban metabolism of food, water, and energy resources. This study conducts a case study of Shihmen Reservoir system, hydropower generation, paddy rice irrigation of Taoyuan and Shihmen Irrigation Associations, and water consumption in Taoyuan, New Taipei, and Hsinchu cities. The interactive influence of the food-water-energy nexus is quantified in this study; the uncertainty analysis of food, water, and energy nexus is presented.

## 1. Introduction

This research aims to analyze food, water, and energy resources of urban metabolism for sustainable resources management. The interactions between the natural environment and humans in cities are complex systems. Urban metabolism is a concept simulating the production, distribution, intake, digestion, and absorption of nutrition, material, energy, and other resources within cities [1,2,3,4,5,6,7,8]. Food, water, and energy are scarce natural resources for human society. The cooperative and competitive relationships are important topics for food-water-energy resources management. The cooperative and competitive relationships of food, water, and energy attract lots of attention; they needed to be quantified [9,10,11,12,13,14,15,16,17].

Previous studies have investigated urban metabolism using life cycle, material flow, meta-analysis, input-output analysis, and simulation tools. The nexus of water, energy, material, carbon dioxide emission, ecological system, climate, and human society has been explored and discussed. Chen and Chen (2017) analyzed the coupling of carbon and energy flows in the cities of Beijing and Issaquah. The research used meta-analysis and nexus modeling to evaluate the energy-carbon nexus [18]. Chen et al. (2018) conducted multi-regional physical input-output analysis for the water-energy nexus in Hong Kong and South China [19]. De Stercke et al. (2018) investigated end-use interactions of the urban water-energy nexus to reduce pressure of water, electricity, and carbon dioxide emissions. The dynamic interactions between London’s water and energy systems was simulated [20]. Facchini et al. (2017) explored the energy metabolisms in 27 of the world’s megacities, including mobile and stationary energy consumption patterns, fuels used, and the end-use and electricity generation mix [21]. Martinez-Hernandez et al. (2017) analyzed water-energy-food and ecosystem interactions. The concomitant Nexus Simulation System (NexSym) is presented to assess a synergistic nexus system in a UK eco-town [22]. Ravalde and Keirstead (2017) compared performance metrics for multi-resource systems of urban metabolism [23]. Schlör et al. (2018) investigated interactions among nature and society of an energy-mineral-society nexus. The nexus is analyzed by the social life cycle assessment model with the scenario based on the Human Development Index [24]. Wang and Chen (2016) addressed the energy–water nexus of urban agglomeration based on multiregional input–output tables and ecological network analysis in the Beijing–Tianjin–Hebei region [25]. Yang et al. (2018) explored the environmental pressures in urban sectors using a energy-water-carbon nexus perspective. This study employed environmental input–output analysis to estimate sectoral embodied energy, water, and CO_2_ in Beijing and Shanghai [26]. Zhang et al. (2018) utilized statistics, equilibrium, econometric, ecological, life cycle, system dynamics, agent-based, and integrated index methods to explore the water-energy-food nexus [27].

**Figure 1:**
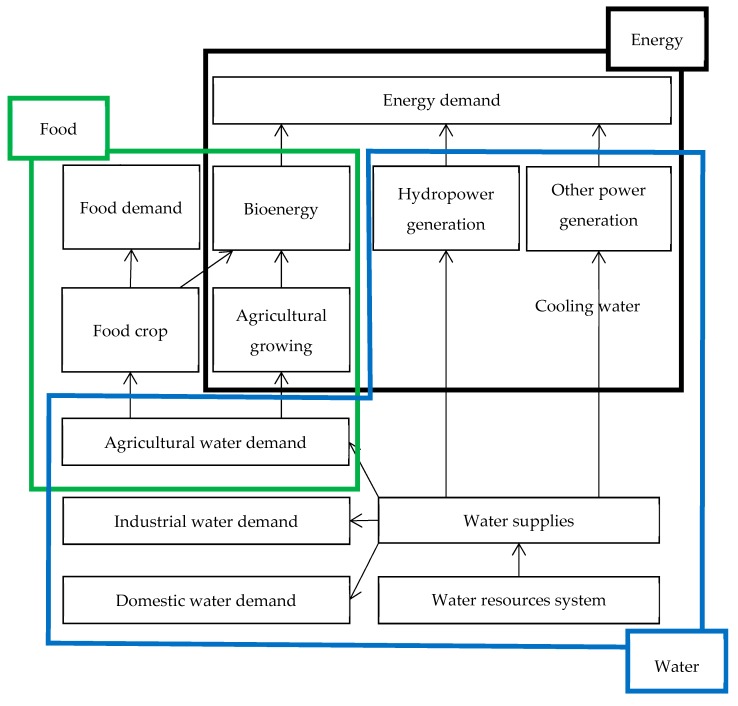
Urban metabolic analysis of food, water, and energy resources systems.

Related studies of the urban metabolism involve large-scale, conceptual, life cycle, or simulation modeling analysis. This research aims to investigate the cooperative/competitive interaction of the water-energy-food nexus. Furthermore, quantitative analysis of the interactive influence of the food-water-energy nexus is presented in this study. A case study in a system with water supply, water consumption, hydropower generation, and paddy rice growing is also conducted. Figure 1 depicts the cooperative/competitive relationship in the food-water-energy nexus for urban metabolic analysis. The Integrated management and tradeoff of water, food, and energy is analyzed in this study.

In this research, system dynamics models are established to analyze the urban metabolic food-water-energy systems. System dynamics was developed in 1950; the simulation method is widely applied in industrial, engineering, natural science, and public policy fields [28,29,30,31,32,33,34,35,36]. Furthermore, system dynamics can be used to examine optimal state trajectories and tradeoffs of systems by solving dynamic differential system equations [37,38,39,40]. Applications of system dynamics and optimization include mechanical, aerospace, chemical engineering, economics, management sciences, and other related fields [38,39,40,41,42].

The system dynamics models are used to examine cooperative/competitive behaviors, including the water supply, water consumption, hydropower generation, and food crops growing in food-water-energy systems. The urban metabolism simulation model is established on NetLogo platform. NetLogo system is a popular and powerful system dynamics simulation and agent-based interactive simulation platform. NetLogo models utilize system dynamics, patch, turtle, plot, and monitor agents to analyze the system performance and agent interactions. In our analysis, NetLogo uses patches and turtle agents to simulate water, paddy rice, hydropower production, and demand/supply interactions. Furthermore, monitor agents collect and display dynamics changes of food, water, and energy nexus systems.

Compared with related studies, the significant contribution of this research includes the establishment of a framework for analyzing food-water-energy nexus, quantifying competition/cooperation among resources, and integrated resources management. In addition, this research constructs system dynamics models for participants in food-water-energy systems. Accordingly, the system dynamics model is established on the NetLogo platform and then equilibrium solutions of competition/cooperation are determined. Moreover, a case study of competition and cooperation among resources is conducted and discussed. This paper is organized as follows. Section 2 formulates system dynamics models to analyze the urban metabolic food-water-energy system. Section 3 presents results and discussion of a case study in Taiwan. The conclusion is addressed in Section 4.

## 2. Materials and Methods

This section begins with formulating a system dynamic model to investigate the food, water, and energy nexus. The model is capable of analyzing complex interactive component behaviors and nonlinear system performance over time. The system dynamics model of the food-water-energy system is established as follows. The water resources network model is formulated including dams, water flow, water consumption, and hydropower production. In the model, water inflow, loss, and spillage at node i are indicated as ${\mathrm{IN}}_{i}^{}$, ${\mathrm{LOSS}}_{i}^{}$, and ${\mathrm{spill}}_{i}^{}$. The storage capacity of node i is ${\mathrm{STORAGE}}_{i}^{}$. ${\mathrm{flow}}_{i,j}^{}$ represents water flow delivered from node i to node j, and ${\mathrm{supply}}_{i,k}^{}$ indicates water supply from node i to demand node k.

The model is a system dynamics simulation model with multiple objectives. Three objective functions are formulated, including food production maximization and energy production maximization. Water resources supply and demand are considered in the constraints. The objective functions are subject to constraints of water mass balance, food yield, hydropower generation, bioenergy production, reservoir operation, and non-negativity equations.

Assuming $R1_{p}^{}$ to be the food production rate of crop p, Equation (1) calculates the food production of crop p. $R2_{p}^{}$ represents the bioenergy production rate and total bioenergy production is estimated in Equation (2). Equation (3) calculates total hydropower generation of the water resources system. Equation (4) establishes a differential equation of water dynamic change, $\frac{{\mathrm{ds}}_{i}^{}\left(t\right)}{\mathrm{dt}}$, considering total upstream inflow ($\overset{}{\underset{j\in \mathrm{up}\left(i\right)}{\sum }}\left(R3_{j,i}^{}\times {\mathrm{flow}}_{j,i}^{}\right)$), total downstream outflow ($\overset{}{\underset{j\in \mathrm{down}\left(i\right)}{\sum }}{\mathrm{flow}}_{i,j}^{}$), loss, supply, and spill at node i. Equation (5) decide crop growing plans of farm I; $x_{i,p}^{}$ is tillage size of crop p with water supply from node i and ${\mathrm{FARM}}_{i}^{}$ is farm size with water supply from node i. In Equation (6), agricultural water demand in node i at time t is $\overset{}{\underset{p}{\sum }}\left(R4_{p}^{}\times x_{i,p}^{}\left(t\right)\right)$ where water demand of crop p is $R4_{p}^{}$. Hydropower production rate at node i is $R5_{i}^{}$ and hydropower generation is calculated in Equation (7). The water supply of ${\mathrm{supply}}_{i,k}^{}\left(t\right)$ for residential, industrial, and agricultural demand of ${\mathrm{waterdmd}}_{i,k}^{}\left(t\right)$ in node i at time t are constructed in Equation (8). The capacity constraint of dam storage $s_{i}^{}\left(t\right)$ is formulated in Equation (9). Non-negativity constraints are established in Equations (10) and (11). The multi-objective system dynamics model in Equations (1)–(11) is applied to simulate and analyze the tradeoff among food, renewable energy production, and water supply.

(1) $${\mathrm{food}}_{p}^{}=\int_{t}^{}\sum_{i}^{}\left(\left(R1_{p}^{}\right)\left(x_{i,p}^{}\left(t\right)\right)\right)\mathrm{dt}$$

(2) $$\mathrm{biofuel}=\int_{t}^{}\sum_{i,p}^{}\left(\left(R2_{p}^{}\right)\left(x_{i,p}^{}\left(t\right)\right)\right)\mathrm{dt}$$

(3) $$\mathrm{hydropower}=\int_{t}^{}\sum_{i}^{}{\mathrm{hydropower}}_{i}^{}\left(t\right)\mathrm{dt}$$

(4) $$\frac{{\mathrm{ds}}_{i}^{}\left(t\right)}{\mathrm{dt}}={\mathrm{IN}}_{i}^{}+\sum_{j\in \mathrm{up}\left(i\right)}^{}\left(\left(R3_{j,i}^{}\right)\left({\mathrm{flow}}_{j,i}^{}\right)\right)-{\mathrm{LOSS}}_{i}^{}-\sum_{j\in \mathrm{down}\left(i\right)}^{}{\mathrm{flow}}_{i,j}^{}-\sum_{k}^{}{\mathrm{supply}}_{i,k}^{}-{\mathrm{spill}}_{i}^{}\;\forall \;i,\;t$$

(5) $$\sum_{p}^{}x_{i,p}^{}\left(t\right)\leq {\mathrm{FARM}}_{i}^{}\;\forall \;i,\;t$$

(6) $${\mathrm{waterdmd}}_{i,k}^{}\left(t\right)=\sum_{p}^{}\left(\left(R4_{p}^{}\right)\left(x_{i,p}^{}\left(t\right)\right)\right)\;\forall \;i,\;k=\mathrm{AG},\;t$$

(7) $${\mathrm{hydropower}}_{i}^{}\left(t\right)=R5_{i}^{}\times \left(\sum_{j\in \mathrm{down}\left(i\right)}^{}{\mathrm{flow}}_{i,j}^{}+\sum_{k}^{}{\mathrm{supply}}_{i,k}^{}+{\mathrm{spill}}_{i}^{}\right)\;\forall \;i,\;t$$

(8) $${\mathrm{supply}}_{i,k}^{}\left(t\right)\geq {\mathrm{waterdmd}}_{i,k}^{}\left(t\right)\;\forall \;i,\;k,\;t$$

(9) $$s_{i}^{}\left(t\right)\leq {\mathrm{STORAGE}}_{i}^{}\;\forall \;i,\;t$$

(10) $$\mathrm{biofuel},{\;\mathrm{flow}}_{i,j}^{},{\;\mathrm{food}}_{p}^{},{\;\mathrm{hydropower}}_{i}^{}\geq 0\;\forall \;i,\;j,\;p,\;t$$

(11) $$s_{i}^{},{\;\mathrm{spill}}_{i}^{},{\;\mathrm{supply}}_{i,k}^{},{\;\mathrm{waterdmd}}_{i,k}^{},{\;x}_{i,p}^{}\geq 0\;\forall \;i,\;j,\;k,\;p,\;t$$

The food-water-energy system dynamics model is a time-dependent simulation problem. The study applies the system dynamics simulation method to analyze the model in Equations (1)–(11). The method simulates control and state variables of the system under different uncertain scenarios.

This research analyzes the system dynamics model of the urban metabolic food-water-energy system in Equations (1)–(11). A system dynamics model is established by using NetLogo to simulate the components interaction and system performance. Different scenarios are simulated and compared by the NetLogo system dynamics model. NetLogo is a simulation modeling platform and is able to conduct system dynamics and agent-based simulations. NetLogo is an open source software widely applied for simulations in physics, chemistry, biology, economics, social science and other related research fields. The model simulates agent-based interaction by using stationary Patch agents and mobile Turtle agents. Additionally, Link agents are used for connecting multiple agents, and Observer agents are set to record individual behavior and system performance.

## 3. Results and Discussion of Urban Metabolic Food-Water-Energy System

**Figure 2:**
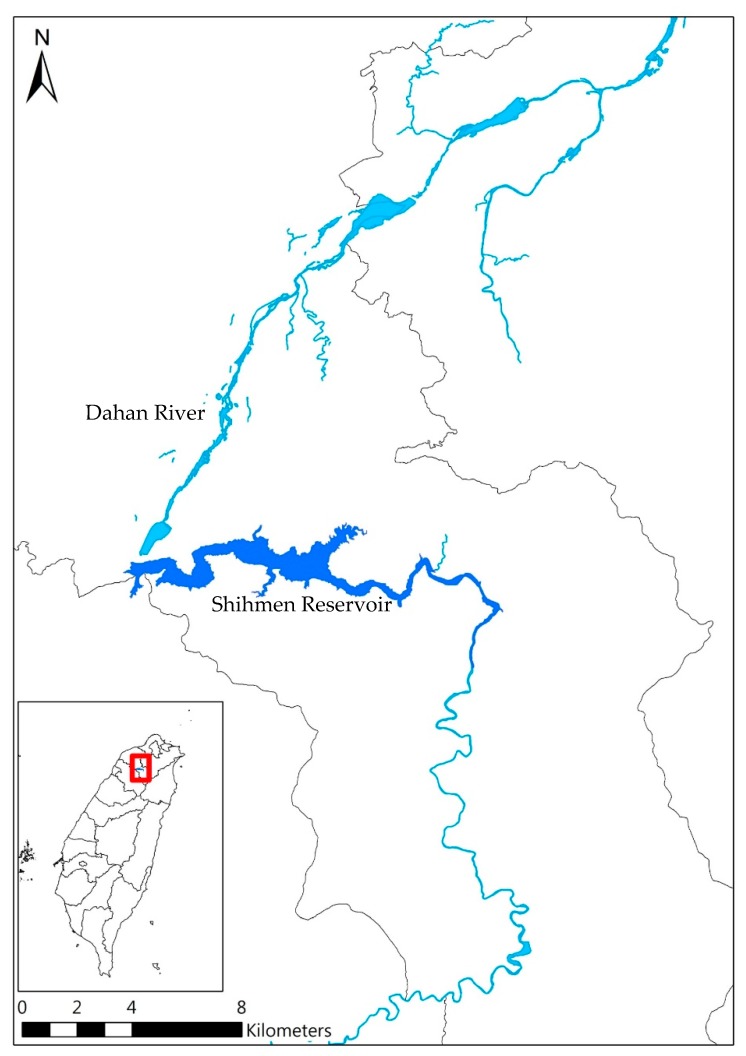
Shihmen Reservoir and Dahan River.

The research conducts a case study of the urban metabolic food-water-energy system in the Shihmen Reservoir system. The food-water-energy nexus system includes the Shihmen Reservoir water resources system, hydropower generation system, paddy rice land, and household water consumption in Taoyuan, New Taipei, and Hsinchu cities (see Figure 2). Accordingly, water supply, water demand, hydropower generation, and paddy rice production are considered and simulated in the system dynamics modeling.

**Figure 3:**
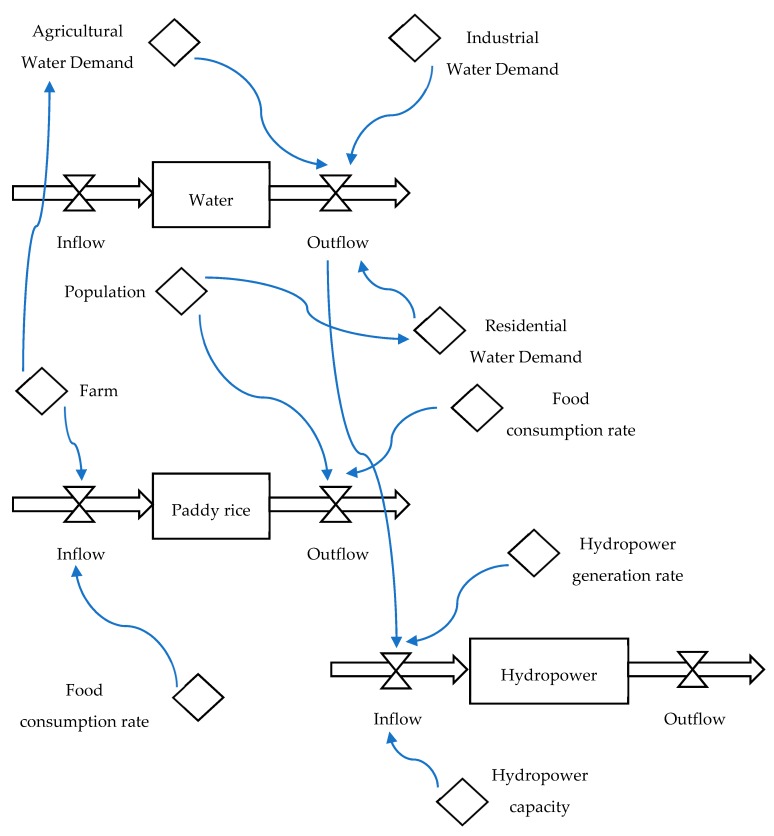
System dynamics simulation of food-water-energy system for urban metabolic analysis.

This research constructs a system dynamics model. The model includes system dynamics simulation and agent-based models. Figure 3 presents the system dynamics simulation of the food-water-energy system for urban metabolic analysis. The system dynamics model simulates time-varying water demand, water supply, food production, hydropower generation, and related resources interactions. In this research, an agent-based model is established to examine the interactive behavior and system performance of food-water-energy. The model displays a multi-agent based NetLogo model with patch, turtle, plot, and monitor agents to analyze the agent interactions. In the multi-agent based NetLogo model, patches are created to simulate water, paddy rice, and hydropower agents. Turtle agents are established to analyze water demand and supply, food production and consumption, and hydropower generation. In addition, monitors and graphs of food, water, and energy are established to collect and display dynamics changes of the systems.

In the simulation, farm land for paddy rice production is about 36,000 hectares in Taoyuan; farm land includes approximately 24,000 hectares of the Taoyuan Irrigation Association and 12,000 hectares of the Shihmen Irrigation Association. Rice production rate is about 5 ton/hectare. For the water supply system, the Shihmen Reservoir is a multi-purpose reservoir located in the upstream of Dahan River in northern Taiwan (Figure 2). It is the third largest reservoir in Taiwan with a storage capacity of 210 million m^3^ and a catchment area of 760 km^2^. The reservoir is confronted with a serious sediment accumulation problem. One-third of the active storage capacity has been taken over by sediments.

In the model, the Shihmen Reservoir supplies water to a population of 3.5 million people in Taoyuan, New Taipei, and Hsinchu cities. The Shihmen Reservoir delivers water to irrigation land for agricultural water consumption of the Taoyuan Irrigation Association and the Shihmen Irrigation Association. Agricultural water demand, on average, accounts for 45% of the total water supply of the Shihmen Reservoir while the rest goes to the domestic (household and industrial) water demand. Furthermore, the Shihmen Reservoir has two hydropower generators, and each of them has a capacity of 45 MW. The hydropower system has a maximal water flow of 140 m^3^/s and total installed capacity of 90 MW. The average annual hydropower generation is about 230,000 MWh. The maximal rice production is estimated at 180,000 tons per season for 36,000 hectares of paddy rice land of the Taoyuan Irrigation Association and the Shihmen Irrigation Association. The average water consumption of paddy rice irrigation is approximately 350 million m^3^.

**Figure 4:**
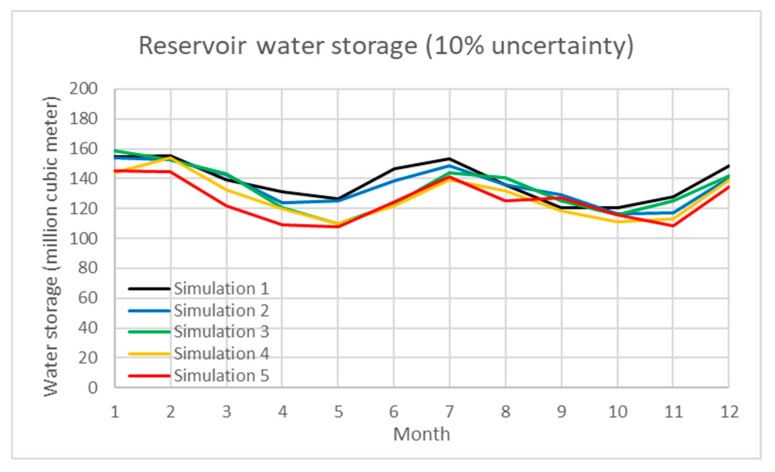
Simulation results of water storage in the Shihmen Reservoir under 10% uncertainty of water inflow.

**Figure 5:**
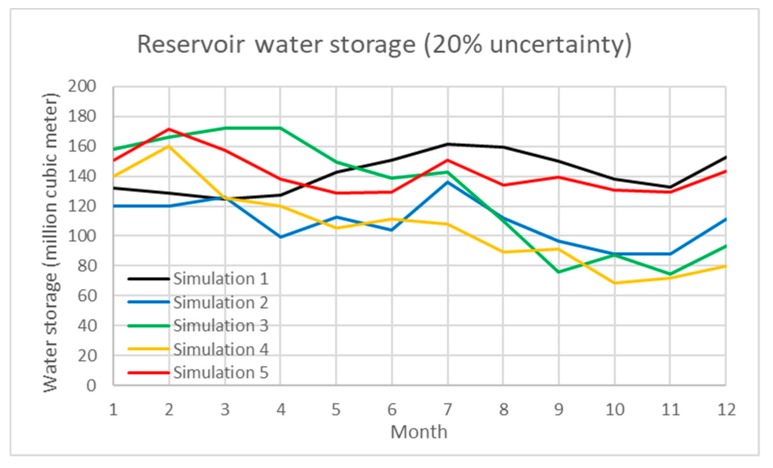
Simulation of water storage in the Shihmen Reservoir under 20% uncertainty of water inflow.

**Figure 6:**
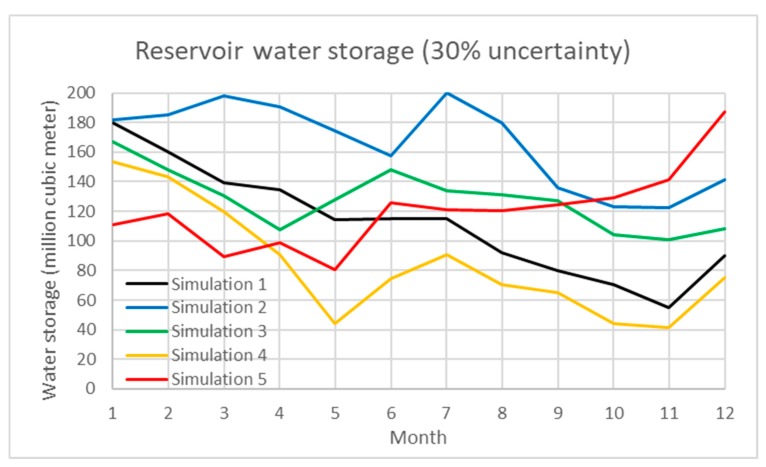
Simulation of water storage in the Shihmen Reservoir under 30% uncertainty of water inflow.

In addition, uncertainty analysis of the water inflow of reservoir, water demand, and paddy rice production are simulated using a Monte Carlo simulation in the model. Table 1 demonstrates the simulation results under various scenarios. The percentage of uncertainty ranges from 5 to 40%. The total water inflow/outflow, average water storage and average generated hydropower under each scenario are shown with the standard deviation. In the results, the interactive influence between water storage and hydropower under uncertain scenarios are also presented. In the study, Scenarios 1 to 3 evaluate the 10, 20, and 30% uncertainty of water inflow of the Shihmen Reservoir and demand. Figure 4, Figure 5 and Figure 6 present the dynamic water storage of the Shihmen Reservoir correspondingly.

## 4. Conclusions

Food, water, and energy are essential and scarce resources. The interactive relationship and allocation of food, water, and energy resources are crucial topics. This research analyzes the integrated food-water-energy systems for urban metabolism. The contribution of this study is to establish a framework for quantifying the cooperation/competition of multiple resources and formulating the system dynamics models of the urban metabolic food-water-energy system. The system dynamics model is capable of simulating different uncertain scenarios for uncertain analysis. A case study of food-water-energy system is conducted consisting of the Shihmen Reservoir water resources system, hydropower generation, paddy rice production of the Taoyuan and Shihmen Irrigation Associations, and domestic water supply in Taoyuan, New Taipei, and Hsinchu cities. The results quantify the interactive influence between food, water, and energy resources. In addition, stochastic simulation of water inflow shows uncertain water supply, paddy rice growing, and hydropower production systems. Future studies include simulation of uncertain climate scenarios, human behavior, hydrological process, energy availability, and energy load of nexus systems. Interactive agent-based modeling with multi-objective in food-water-energy systems are also of interest.

## Figures and Tables

**Table 1:** Uncertain simulation of water inflow, outflow, storage, and hydropower of the Shihmen Reservoir.

Uncertainty	Water Inflow	Water Outflow	Water Storage	Hydropower
	Total (Standard Deviation)	Total (Standard Deviation)	Mean (Standard Deviation)	Mean (Standard Deviation)
	million m^3^	million m^3^	million m^3^	MWh
5%	776.13	773.61	124.82	322,338
	(4.92)	(15.60)	(15.57)	(6499)
10%	785.45	789.23	133.36	328,846
	(5.35)	(17.72)	(19.81)	(7384)
15%	817.02	787.63	150.95	328,181
	(7.10)	(19.17)	(15.30)	(7988)
20%	764.54	754.18	135.54	314,241
	(5.82)	(14.16)	(13.06)	(5900)
25%	759.71	820.12	59.62	341,717
	(12.27)	(22.75)	(36.85)	(9479)
30%	763.26	776.79	108.59	323,662
	(12.55)	(16.74)	(21.53)	(6975)
35%	759.14	759.88	143.83	316,616
	(16.49)	(18.18)	(19.40)	(7576)
40%	834.72	754.82	121.33	314,510
	(15.68)	(24.33)	(39.35)	(10,136)

## Nomenclature

In this paper, lowercase letters are used to indicate indices and decision variables, and uppercase letters indicate given parameters. Indices, given parameters, and variables are listed below and followed by the definitions.

*Indices*	
i, j	node of water network, i, j=1, 2, 3, …, I
k	residential, industrial, and agricultural water demand, i=RES, IND, AG
p	crop, p=1, 2, 3, …, P
t	time, t=1, 2, 3, …, T
*Parameters*	
${\mathrm{FARM}}_{i}^{}$	farm size with water supply from node i
${\mathrm{IN}}_{i}^{}$	water inflow at node i
${\mathrm{LOSS}}_{i}^{}$	water loss at node i
$R1_{p}^{}$	production rate of crop p
$R2_{p}^{}$	biofuel production rate of crop p
$R3_{j,i}^{}$	water loss rate from node j to node i
$R4_{p}^{}$	water demand rate of crop p
$R5_{i}^{}$	hydropower production rate of node i
${\mathrm{STORAGE}}_{i}^{}$	water storage capacity at node i
*Variables*	
biofuel	biofuel production
${\mathrm{flow}}_{i,j}^{}$	water flow from node i to node j
${\mathrm{food}}_{p}^{}$	food production of crop p
${\mathrm{hydropower}}_{i}^{}$	hydropower generation at node i
$s_{i}^{}$	water storage at node i
${\mathrm{spill}}_{i}^{}$	water spillage at node i
${\mathrm{supply}}_{i,k}^{}$	water supply of i to demand k
${\mathrm{waterdmd}}_{i,k}^{}$	water demand k supplied from node i
$x_{i,p}^{}$	tillage size of crop p with water supply from node i
